# Subacute hematogenous osteomyelitis of the fibula

**DOI:** 10.11604/pamj.2020.37.236.18004

**Published:** 2020-11-13

**Authors:** Zied Jlalia, Dhia Kaffel

**Affiliations:** 1 Pediatric Orthopedics Department, Kassab Institute of Orthopedic Surgery, Ksar Said, Tunisia,; 2 Rheumatology Department, Kassab Institute of Orthopedic Surgery, Ksar Said, Tunisia

**Keywords:** Child, fibula, anti-bacterial agents, palpation

## Image in medicine

**Figure 1:**
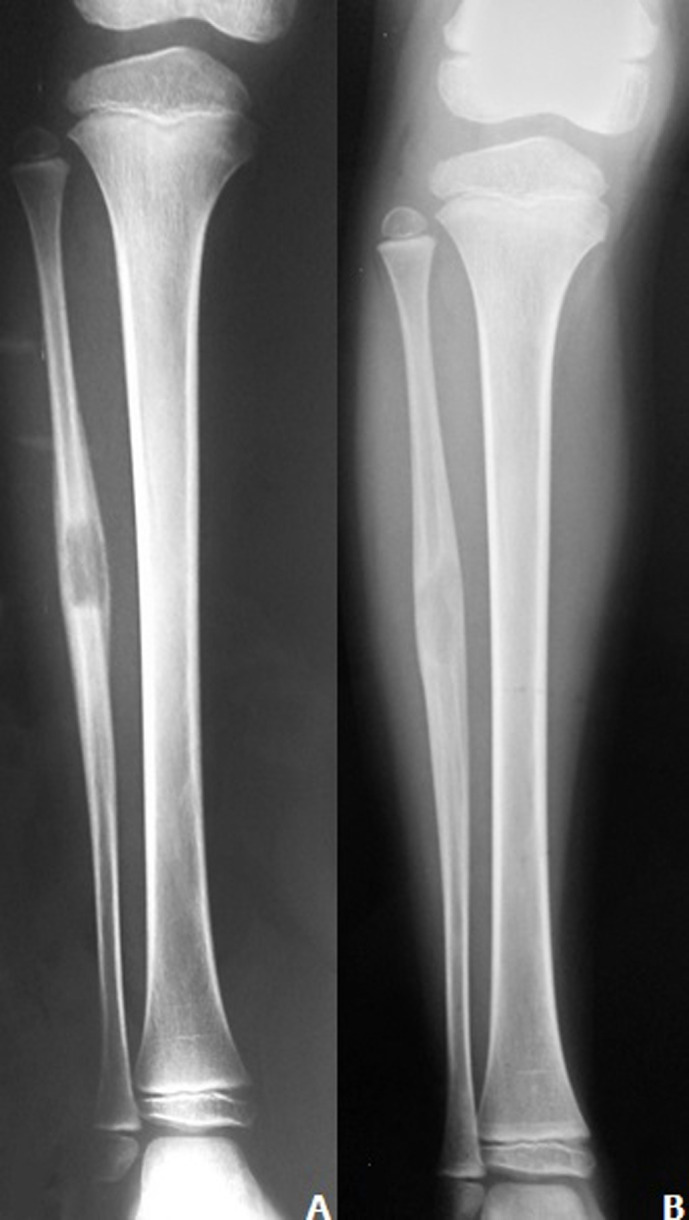
periostal reaction, partial lysis and bone interruption of the fibula (A,B)

A ten years old patient consulted us for lower right limb pain and fever (38.2°C). There were inflammatory signs and palpation pain in relation to the right fibula. Biology was disturbed with white blood cells at 17.470 el/mm^3^, a sedimentation rate at 50 mm/1 hour and a C-reactive protein at 20 mg/l. X-ray shows periostal reaction, partial lysis and bone interruption of the fibula (Figure 1A). Anatomopathological study shows a sclerotic bone and fibrous tissue with altered neutrophils. The diagnosis of subacute osteomyelitis was retained, the patient was put on an oral antibiotic for 21 days and immobilized by a Plaster. X-ray of the fibula after 3 months follow-up shows a bone repair ad-integrum (Figure 1B). In recent years the face of osteomyelitis has changed considerably. The clinic as well as biology are becoming less noisy, probably in relation to the abusive use of antibiotics. The pain was a constant sign, the symptomatology can sometimes be summed up with a refusal of support on the algic part of the skeleton as it was the case for our patient. Local inflammatory signs are sometimes noted. The absence of general signs of infection is constant. It must be evoked before a non-specific radiographic image often metaphyseal, integrated with clinical and biological data.

